# Glucose 6 Phosphate Dehydrogenase (G6PD) quantitation using biosensors at the point of first contact: a mixed method study in Cambodia

**DOI:** 10.1186/s12936-022-04300-9

**Published:** 2022-10-04

**Authors:** Bipin Adhikari, Rupam Tripura, Lek Dysoley, James J. Callery, Thomas J. Peto, Chhoeun Heng, Thy Vanda, Ou Simvieng, Sarah Cassidy-Seyoum, Benedikt Ley, Kamala Thriemer, Arjen M. Dondorp, Lorenz von Seidlein

**Affiliations:** 1 Mahidol‐Oxford Tropical Medicine Research Unit, Faculty of Tropical Medicine, Mahidol University, Bangkok, Thailand; 2 Centre for Tropical Medicine and Global Health, Nuffield Department of Clinical Medicine, University of Oxford, Oxford, UK; 3 C.N.M National Center for Parasitology, Entomology and Malaria Control, Phnom Penh, Cambodia; 4 Global and Tropical Health Division, Menzies School of Health Research and Charles Darwin University, Darwin, Australia

**Keywords:** Village malaria workers, Community, Vivax malaria, G6PD, Quantitative, Radical cure, Cambodia

## Abstract

**Background:**

Quantitative measurement of Glucose-6-Phosphate Dehydrogenase (G6PD) enzyme activity is critical to decide on appropriate treatment and provision of radical cure regimens for vivax malaria. Biosensors are point-of-care semi-quantitative analysers that measure G6PD enzyme activity. The main objective of this study was to evaluate the operational aspects of biosensor deployment in the hands of village malaria workers (VMWs) in Cambodia over a year.

**Methods:**

Following initial orientation and training at Kravanh Referral Hospital, each VMW (n = 28) and laboratory technician (n = 5) was provided a biosensor (STANDARD SD Biosensor, Republic of Korea) with supplies for routine use. Over the next 12 months VMWs convened every month for refresher training, to collect supplies, and to recalibrate and test their biosensors. A quantitative self-administered questionnaire was used to assess the skills necessary to use the biosensor after the initial training. Subsequently, VMWs were visited at their location of work for field observation and evaluation using an observer-administered questionnaire. All quantitative questionnaire-based data were analysed descriptively. Semi-structured interviews (SSIs) were conducted among all participants to explore their experience and practicalities of using the biosensor in the field. SSIs were transcribed and translated into English and underwent thematic analysis.

**Results:**

A total of 33 participants completed the training and subsequently used the biosensor in the community. Quantitative assessments demonstrated progressive improvement in skills using the biosensor. VMWs expressed confidence and enthusiasm to use biosensors in their routine work. Providing G6PD testing at the point of first contact avoids a multitude of barriers patients have to overcome when travelling to health centres for G6PD testing and radical cure. Deploying biosensors in routine work of VMWs was also considered an opportunity to expand and strengthen the role of VMWs as health care providers in the community. VMWs reported practical concerns related to the use of biosensor such as difficulty in using two pipettes, difficulty in extracting the code chip from the machine, and the narrow base of buffer tube.

**Conclusions:**

VMWs considered the biosensor a practical and beneficial tool in their routine work. Providing VMWs with biosensors can be considered when followed by appropriate training and regular supervision. Providing community management of vivax malaria at the point of first contact could be key for elimination.

**Supplementary Information:**

The online version contains supplementary material available at 10.1186/s12936-022-04300-9.

## Background

Countries in the Greater Mekong Subregion (GMS) are committed to eliminating malaria by 2030 [1]. The national malaria control programme of Cambodia under the supervision of National Center for Parasitology, Entomology and Malaria Control (CNM) plans to eliminate malaria already by 2025 [2]. Achieving this ambitious goal presents challenges, particularly the high prevalence of *Plasmodium vivax*, which can relapse years after an initial infection [3, 4].

Despite a decline in the overall malaria burden, the relative increase in vivax malaria remains a major challenge for malaria control programmes in the GMS [5]. Approximately 90% of all malaria in Cambodia is currently caused by *P. vivax*. A total of 9234 vivax malaria cases were reported in Cambodia in 2020 [6, 7]. Due to this relative increase in vivax malaria cases in recent years CNM has enhanced its *P. vivax* specific strategies [2]. To prevent relapse, vivax malaria elimination requires the clearing of hypnozoites with a ‘radical cure’ that includes 8-aminoquinolones [3]. All members of this class of drugs can cause haemolysis in individuals with low activity of the Glucose-6-Phosphate Dehydrogenase (G6PD) enzyme, usually referred to as G6PD deficiency [8]. A previous study in Cambodia found the prevalence of G6PD deficiency among females to be 13.8% and among males between 12.6 and 18.8% [9, 10]. Since the first quarter of 2021, G6PD screening is carried out at health centre level before providing radical cure to vivax patients [6, 7].

G6PD is a cytoplasmic enzyme essential for red blood cells (RBCs) to withstand oxidative stress [11]. The G6PD gene is located on the X chromosome and is thus present as a single copy in males [12]. In heterozygous females, haematopoietic cells produce both G6PD-deficient and G6PD-normal RBCs, resulting in two distinct RBC populations, a deficient and a G6PD normal one. The proportion of both RBC populations varies by individual. Phenotypic G6PD activity in heterozygous females therefore varies from almost no activity to normal activity, with the majority in between (intermediate). Qualitative rapid diagnostic test (RDT) kits differentiate categorically normal versus deficient G6PD enzyme activity overlooking heterozygote females with intermediate enzyme activity. Patients with intermediate enzyme activity are at risk of receiving a G6PD normal qualitative test result despite an increased risk of drug induced haemolysis [13]. The risk of haemolysis in patients with intermediate and deficient G6PD status is high when receiving single dose tafenoquine which has a long half-life of 15 days. Tafenoquine administration thus requires quantitative testing [14].

The quantitative reference method to measure G6PD activity is spectrophotometry but is not suitable for point of care testing as it requires trained laboratory technicians and most critically a functioning laboratory [15–18]. Over the last years, handheld devices, specifically biosensors have been introduced some of which offer better operational features than spectrophotometry at a lower price, with performance at least under research conditions suitable for routine use [19]. One of these devices, the STANDARD biosensor (SD Biosensor, South Korea) is currently piloted in laboratory-based studies [20]. The biosensor has also been piloted in clinical trials, and implementation studies but the outcome on their accuracy and reliability is yet to be published. In laboratory-based studies the biosensor has been found to be reliable [21] and operationally appropriate when tested by laboratory technicians and health workers after training in laboratory facilities [22–24].

As malaria transmission recedes to more remote areas, VMWs (village malaria workers) and MMWs (mobile malaria workers), here collectively referred to as VMWs are the first and often the only point of contact with the health system [25, 26]. In such remote communities, VMWs are essential for the provision of health services. To ensure that all patients with vivax malaria have access to radical cure, an appropriate 8-aminoquinoline regimen has to be provided and, therefore, G6PD testing should be conducted at the point of first contact.

The main objective of the roll out radical cure (RORC) research project was to explore the feasibility and acceptability of biosensor use by VMWs in rural Cambodian villages. Biosensors were provided to VMWs and health centre-based lab technicians after training to perform G6PD tests among vivax malaria and febrile patients over the period of a year. Up to now, no studies have explored the long-term community-based use, acceptability, and practicalities of deploying biosensors in the hands of VMWs [21–24].

## Methods

### An overview of roll out radical cure (RORC)

**Fig. 1:**
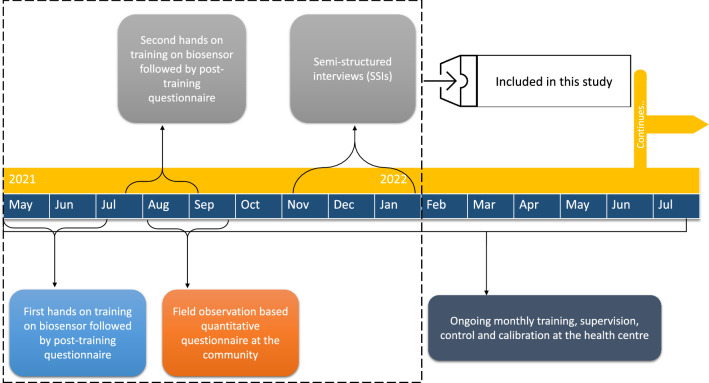
Schematic diagram of study activities of RORC

RORC was an operational study [27] conducted in villages within Kravanh district, Pursat province, Cambodia over a year in which VMWs were trained and provided with the biosensors (Fig. 1). In this study, a mix of various methods was used to answer the research question. The study incorporated quantitative questionnaires, observations, reports and semi-structured interviews. The study followed a qualitative-dominant, mixed methods research [28, 29].

A total of 33 individuals (28 VMWs and 5 laboratory technicians) were enrolled for orientation and training on biosensors between May and July 2021. All VMWs working under the oversight of Kravanh and Prognil health centres were contacted and were enrolled based on their active status in malaria case management at the community and interest to be involved with the RORC. Because of the COVID-19 pandemic, VMWs and laboratory technicians were trained in small groups at various time points (each group consisted of 5 to 6 persons). All orientation and training workshops were conducted under the supervision of a senior lab technician (OS) who was trained by an expert in biosensor use (BL) and was also responsible for training fellow lab technicians and study coordinators (CH and TV).

**Fig. 2:**
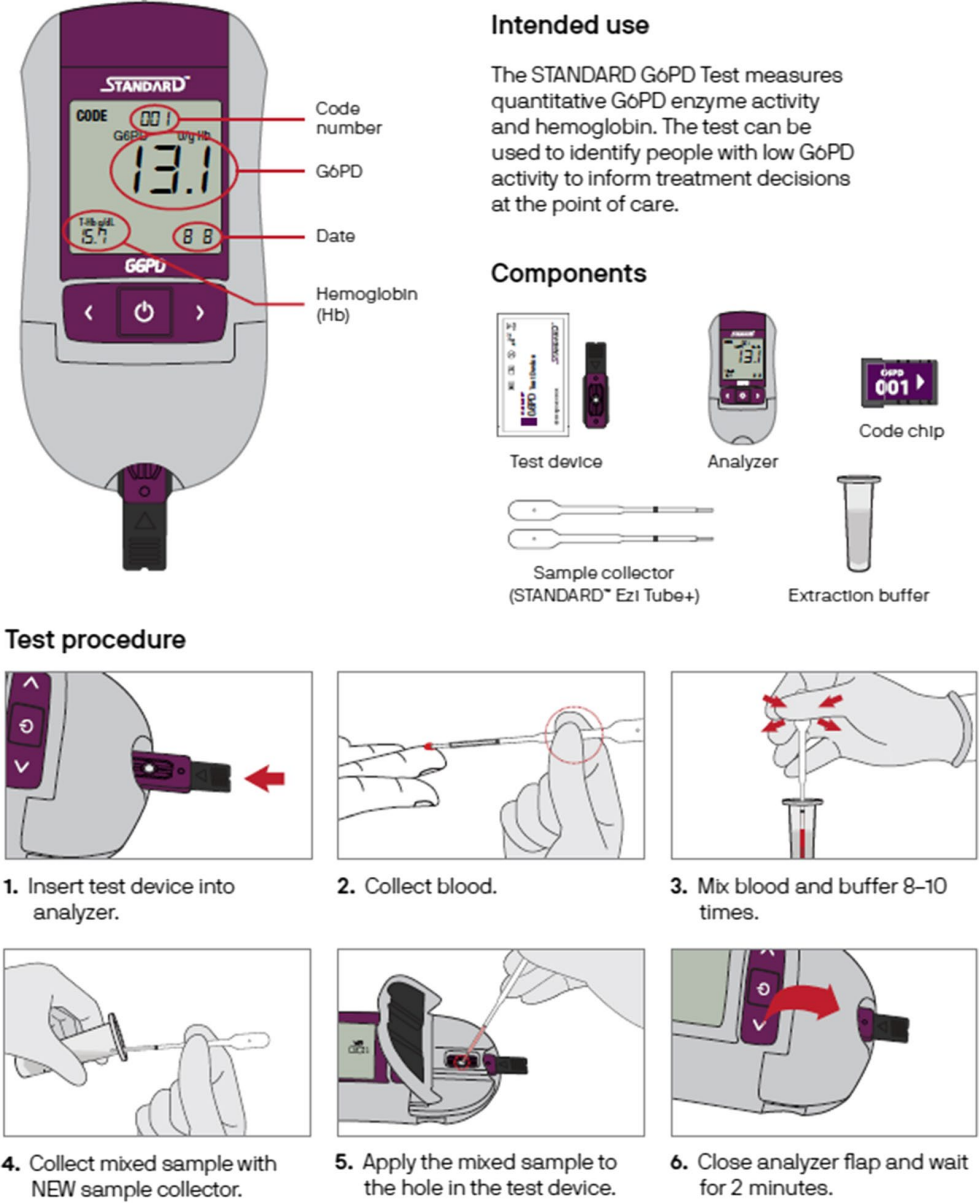
Stepwise illustration of G6PD test using biosensor (reproduced from the PATH website at www.path.org dated 28 June 2022)

Each training entailed a 1 day workshop that included background information on *P. vivax*, radical cure, the role of the G6PD enzyme, the use of the biosensor and interpretation of its results. The training was followed by a practical session supervised and supported by two study coordinators in addition to biosensor users and laboratory technicians. Participants were encouraged to repeat tests (two to three in each session) to ensure procedural steps were completed correctly and smoothly. Stepwise illustration of how G6PD measurement is done using biosensor was part of the training (Fig. 2).

Following the initial training, all VMWs and laboratory technicians remained in regular contact with the study coordinators (n = 2) via phone. To practice using the biosensors, each VMWs were provided the biosensor and sufficient supplies to measure G6PD activity among vivax and febrile (non-malaria) patients. The G6PD activity of 1077 febrile and 63 vivax patients was tested by 28 VMWs over the study period. Concerns related to the use and interpretation of the biosensor results were discussed with the trainers as they occurred. In addition, trainers convened VMWs every month for calibration, and quality control of the biosensors.

### Quantitative questionnaire

A total of two training sessions for each VMW were conducted a month apart (day 0/T0 and day 30/T30). After the end of the hands-on training sessions (both T0 and at T30), all attendees completed a self-administered questionnaire that assessed their knowledge and skills related to use of the biosensor and the interpretation of the results. The self-administered questionnaires were developed by PATH (Additional file 1). The questionnaire was developed in line with the recommendations outlined by the US Food and Drug Administration [30]. The questionnaire assesses end user proficiency of the biosensor and fulfils data requirements from the WHO prequalification technical specification series TSS-2 for in vitro diagnostics medical devices [31]. The questionnaire was used in a recent multi-country study to assess biosensor user proficiency [22].

### Observation based quantitative questionnaire at the community setting

Observation-based assessments were carried out after T30 between the 4th and 5th months (August and September) by two of the trainers. Based on prior discussions, each VMW made an appointment with a patient so that the trainers can observe procedural steps followed by VMWs. Trainers assessed how VMWs used the machine by applying a questionnaire that was modified from the tool developed by FIND [version 1.0 dated 2 October 2019] (Additional file 2) designed to assess the practical proficiency of using biosensor at the end-user’s setting, which in this study is the community [32].

Both questionnaires (Additional files 1 and 2) were translated into Khmer by a professional translation service. All translated items of the questionnaire were cross-checked with the English version by bi-lingual study team members (CH, TV) to ensure they were translated correctly. Translated versions of the questionnaire were tested among four health workers (non-participants) to ensure they were clear and comprehensive. Minor adjustments were made based on the feedback. All data from these questionnaires were descriptively analysed.

### Semi-structured interviews (SSIs)

SSIs were conducted among VMWs and lab technicians who participated as biosensor users in this study (Table 1). SSIs were designed to explore the practicalities of using the biosensors by VMWs and lab technicians (Additional file 3). The overall aim of the SSIs was to explore individuals’ experience with the biosensor, how it fits into their daily work routine, technical and practical issues encountered, ways to improve the machine, and end-user opinions on whether the biosensor is suitable for use in local communities. All SSIs were conducted in field settings following an appointment with each respondent between November 2021 and January 2022. SSIs were conducted during the second half of the study to document their experience and practicalities of using the biosensor. Two trainers (CH and TV) conducted the interviews in the local language, Khmer. All interviews were audio-recorded and later transcribed and translated into English language by the interviewers.

**Table 1:** Socio-demographics of participants of Semi-Structured Interviews (SSIs)

SSI	Age (years)	Sex	Qualification (years of education)
SSI-1	65	M	7
SSI-2	55	F	7
SSI-3	30	F	12
SSI-4	42	F	6
SSI-5	51	F	6
SSI-6	40	F	9
SSI-7	38	F	12
SSI-8	54	F	12
SSI-9	41	F	9
SSI-10	59	F	9
SSI-11	27	M	12
SSI-12	23	M	12
SSI-13	41	M	9
SSI-14	43	M	7
SSI-15	41	F	7
SSI-16	50	F	6
SSI-17	51	M	8
SSI-18	54	M	9
SSI-19	49	F	9
SSI-20	36	F	5
SSI-21	30	F	6
SSI-22	25	F	7
SSI-23	36	F	7
SSI-24	42	M	9
SSI-25	52	M	12
SSI-26	54	M	12
SSI-27	26	M	University
SSI-28	50	M	12
SSI-29	31	F	6
SSI-30	43	M	9
SSI-31	48	M	4
SSI-32	48	M	11
SSI-33	52	M	2

Information on their workplace is deliberately avoided in the table to prevent potential identification of the participants

All interviews were analyzed following a mix of deductive and inductive approaches using qualitative data management software: NVivo version 12 by QSR international, Australia. Initial themes were derived from a thematic guide/interview guide aligned to the research question (deductive approach). Emerging themes were developed based on the line-by-line coding of data (inductive approach). Findings were discussed among the core team members (who interacted with the participants) and were refined to synthesize the final themes that forms the basis of the result section below. Relevant quotes and interpretations were included to support the themes in the study.

### Dissemination of findings to participants

Dissemination of findings to participants was utilized as a part of post-study engagement in line with the ethical principles for global health research [33]. A total of two informal discussions were held with VMWs that represented each health centre (Prognil and Kravanh). The first meeting was with seven VMWs from Prognil health centre catchment area on 15 June 2022. The second meeting was held on the 21st of June and was attended by nine VMWs from Kravanh Health Centre catchment area. In both meetings, findings from this study were shared and participants were asked for their opinion. All participants approved the findings, and additional information was added to the text.

## Results

**Fig. 3:**
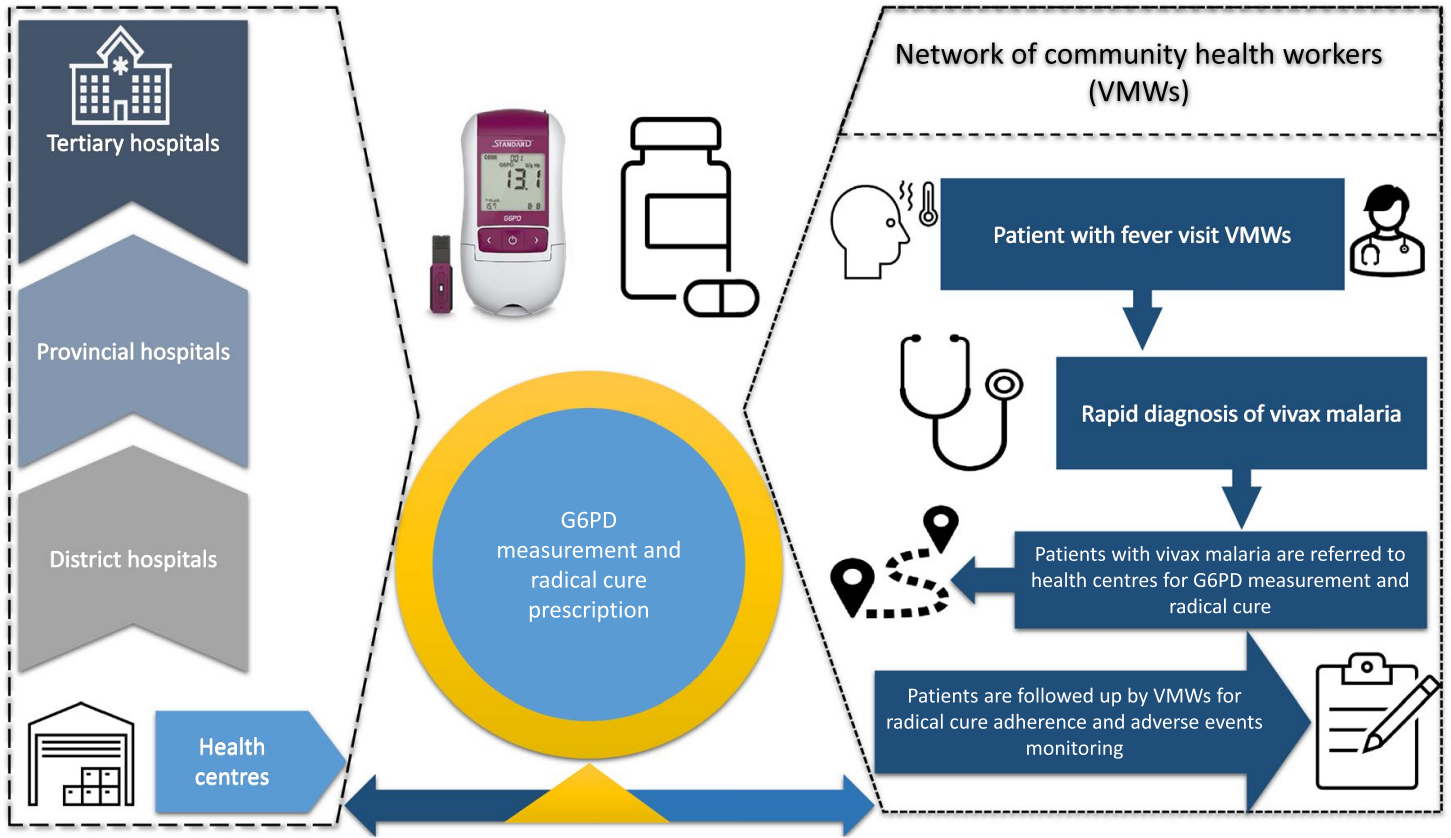
Current scenario of vivax management referral system in Cambodia

**Fig. 4:**
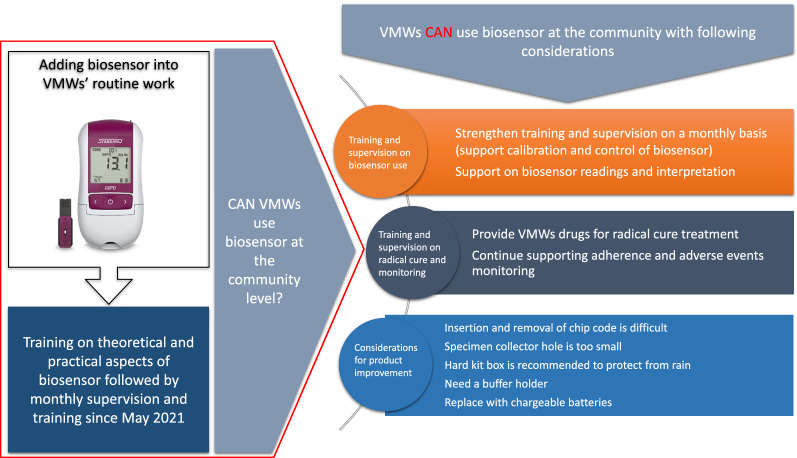
Considerations for integrating biosensor into VMW/MMWs’ routine work at the community

Prior to and during the course of the study, VMWs were referring vivax patients to the health centre (or higher-level health facilities) for G6PD test and prescription of a radical cure regimen (Fig. 3). As a part of this study, after receiving training, support and a biosensor, VMWs reported their enthusiasm about using biosensors in the community and did not perceive integrating its use into their routine work as an additional burden. VMWs appreciated the time and availability of tests to practice that progressively build their skills and confidence in the use of the machine and interpreting the results. VMWs reported constraints related to the use of biosensor for example the use of two pipettes was difficult, black lines in the pipette were poorly visible, the sample placement hole (sample well) in the test device was small, inserting and removing chip code was difficult, and the base of the buffer tube was small making it unstable. Nonetheless, VMWs recommended its deployment at the community level for G6PD testing, interpretation, and potential use (programmatic implementation) for prescribing the radical cure in the community (Fig. 4).

### Characteristics of VMWs’ work prior to the study

**Fig. 5:**
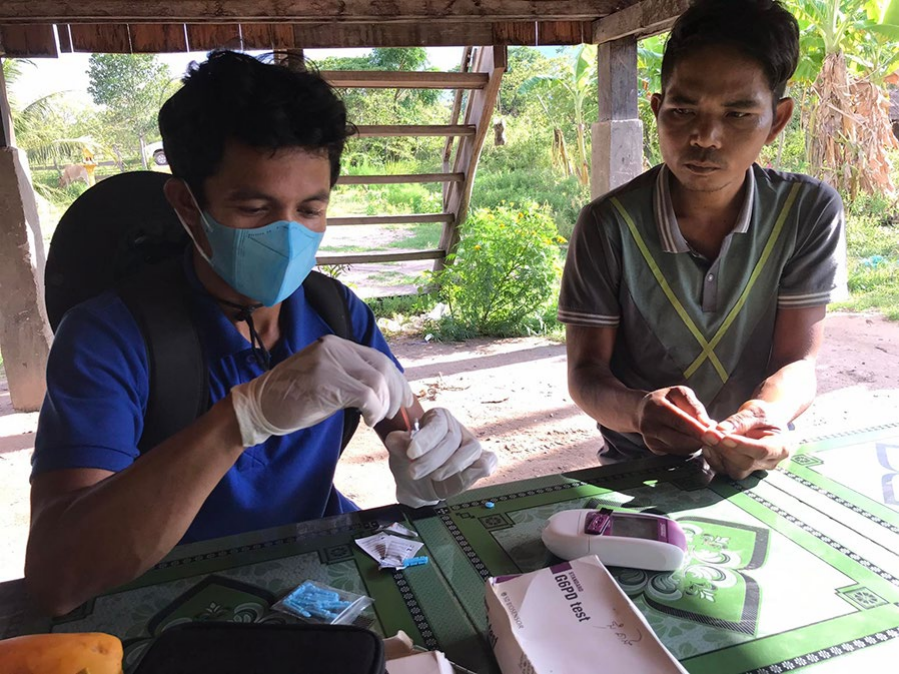
A VMW using a biosensor machine with a patient at the community

VMWs are responsible for diagnosing and managing uncomplicated malaria cases (Fig. 5). Over the last months and years, VMWs have been all too aware of the declining number of falciparum and vivax malaria cases. Prior to the current study, most VMWs were unfamiliar with the quantitative G6PD tests, although a few had an idea about rapid diagnostic tests for G6PD enzyme. Several VMWs had used rapid qualitative diagnostic tests (with binary outcomes), which they referred to as ‘purple test’ under a study conducted by ‘Health and Social Development’ (HSD).

*I: I want to know your daily work, before having this machine for use, how was your work?*

*R: It was hard work because we did not have [this] machine for use, but we had a kind of purple test to see, …… also difficult to work.*

*I: The purple test? what is it looking for?*

*R: To see G6PD too. The test to see colour, it was called SD*

*I: It was SD?*

*R: Yes… it looks like the rapid test.*

*I: Test and see the colour*

*SSI-11*

Prior to the introduction of radical cure treatment for vivax malaria patients, VMWs treated vivax malaria using artemisinin combination therapies, as they do for falciparum malaria cases. Based on the government treatment guidelines beginning from the first quarter of 2021, newly diagnosed vivax malaria patients seeking care from VMWs were offered a first dose of standard anti-malarials and were referred to the health centre for G6PD testing and radical cure treatment (Fig. 3). In the health centres these patients were tested for their G6PD enzyme levels, provided treatment based on G6PD status, and sent home to be followed up by VMWs to assure adherence to the radical cure treatment and monitoring of clinical symptoms on days 3, 7 and 14. Follow-up was reported through a CNM developed app available on their mobile phones.

*I: How do you normally treat vivax malaria patients?*

*R: At that time if a patient came for testing with malaria, and after the test result positive, I gave him 1 dose of AMQ [antimalarial therapy, artesunate/mefloquine] then I sent him to health centre to check G6PD with machine. If his Hb was enough, the primaquine had to be given for 14 days, given the first at health centre, the drugs given to patient, but VMW had to follow-up at home……three time follow-up within 14 days….the follow -up was on day 4, day 7, and day 14.*

*SSI-6*

### Training followed by addition of biosensor into VMWs’ routine work

After the completion of initial training, all 33 participants took part in a post-training assessment using a self-administered questionnaire. Most items in the questionnaire were answered correctly by the participants, although improvements were not consistent in repeat assessment during the second month (Table 2). Items related to reading of G6PD and haemoglobin results were satisfactory (e.g., G6PD 4.5 was read correctly by 100% of participants at both T0 and T30). These questionnaires were utilized by training supervisors to prioritize and focus the areas that were needed during the refresher training and in their routine practice.

**Table 2:** The standard G6PD test assessment among the participants at T0 and T30 (n = 33)

Items	T0	T30
	Number (%)	Number (%)
The STANDARD G6PD test identifies G6PD deficiency	32 (97)	33 (100)
Standard G6PD measures enzyme reaction	24 (73)	32 (97)
Optimal temperature for biosensor operation 15–40 °C	30 (91)	26 (79)
Type of samples for G6PD test: capillary and venous blood	22 (67)	23 (70)
Must matching of chip and strip pouch	26 (79)	25 (76)
Insert the test strip after the code chip is entered into the analyzer	21 (64)	19 (58)
Mix blood and buffer in EziTube 8–10 times	26 (79)	30 (91)
Use 10 µl or the black line on the EziTube	29 (88)	28 (85)
Apply the mixture immediately to the test strip	11 (33)	0
Number of Ezi tubes to run the samples = 2	26 (79)	26 (79)
Test strip cannot be reused	30 (91)	33 (100)
Correct precautions to avoid the injury	14 (42)	17 (52)
G6PD 1.2 is deficient	27 (82)	27 (82)
G6Pd 13.1 is read correct	33 (100)	33 (100)
Hb 15.7 is read correct	31 (94)	32 (97)
G6PD 13.1 is normal	29 (88)	31 (94)
G6PD 0.7 is read correct	33 (100)	33 (100)
Hb 11.1 is read correct	32 (97)	33 (100)
G6PD 0.7 is deficient	33 (100)	32 (97)
G6PD 4.5 is read correct	33 (100)	33 (100)
Hb 13.4 is read correct	32 (97)	32 (97)
G6PD 4.5 is intermediate	29 (88)	25 (76)
G6PD n-A is read correct	32 (97)	33 (100)
Hb Lo is read correct	32 (97)	33 (100)
Test did not work (error or NA)	33 (100)	33 (100)
G6PD 2.0 is read correct	33 (100)	33 (100)
Hb 13.2 is read correct	33 (100)	33 (100)
G6PD 2.0 is deficient	33 (100)	33 (100)
G6PD E-2 is read correct	32 (97)	31 (94)
Hb (none)	33 (100)	33 (100)
Test did not work (error or NA)	30 (91)	33 (100)
G6PD 9.2 is read correct	32 (97)	33 (100)
Hb 5.8 is read correct	31 (94)	33 (100)
G6PD 9.2 is normal	27 (82)	19 (58)

VMWs in this study reported a strongly positive experience using biosensors in their routine work. They expressed the opinion that biosensors could aid in their routine responsibilities, particularly for vivax malaria management in the community. None experienced the use of the biosensor as a burden, rather attempted to rationalize its need in their routine work.

*I: Overall, do you find biosensors well integrated in your work? (have you accepted them [biosensors] or are they still burden in your work?) Why?*

*R: Yes, it is well integrated because it is not different part of work. When I do malaria test, it is the same part with my work.*

*I: Have you ever thought that this machine was difficult to use? it should be used at health centre?*

*R: No. At [the] beginning, when it [a patient] needed treatment with radical cure, I detected vivax and I sent him to health centre, and G6PD was checked by health centre staff. I was not allowed [to treat], and radical cure has been treated [provided] by health centre, but now I have machine, I can know whether the radical cure will be used [needed].*

*I: Oh… we can that.*

*R: and it is not difficult, it is easy.*

*SSI-10*

In the context of declining malaria, expanding VMWs’ capacity by training and providing them with the biosensors promoted their perceived sense of roles and responsibilities in the communities. Apart from gaining the skill sets required to use the biosensors, VMWs also expressed their satisfaction while undertaking these responsibilities, specifically because they were now able to inform patients about their G6PD enzyme activity and haemoglobin levels. Importantly, such responsibilities helped them sustain their relationship and build trust with their community members.

*I: Overall, do you find biosensors (well) integrated in your work? (have you accepted them or are they still burden in your work?) Why?*

*R: I think it is integrating for volunteer [referring to themselves or VMWs].*

*I: Do you think it is burden?*

*R: No…it is not [a] burden, because I work for that job and this is given, I am so happy.*

*I: Can you explain me why you are happy?*

*R: I can check up with people, and my villagers trust me and say that I provide good treatment to them, it is a kind of building trust as well. I [do] everything for people’s health, and it is useful for people.*

*SSI-15*

In response to whether these additional tests were a burden to patients, participants simply asked about the purpose of the test out of curiosity (but not with reluctance to be tested) as it was apparently a new machine to them.

*R: They asked what the purpose of checking with that machine.*

*I: Most of them asked that question?*

*R: Yes, and I told it checks enzyme in your body; if it is low, we cannot treat that malaria with radical cure, if enzyme is enough it can be treated for radical cure, and it won’t relapse if mosquito won’t bite again.*

*I: Did patients complain about sample that we took?*

*R: No… because we tested for malaria and the same time, we tested for G6PD [with the same sample]. No problem with that small amount of blood….to avoid pricking few times.*

*SSI-10*

VMWs also received feedback from patients regarding the use of the biosensor, particularly if such machines could diagnose other diseases such as typhoid fever and stomach problems.

When asked about whether these machines should be deployed at health centre or at VMW level, VMWs expressed their preference of using biosensors in the community thus mitigating multiple barriers that patients have to overcome by seeking health care at health centres or hospitals.

*I: Do you think this machine should be used at health centre or hospital or at VMWs? which one is better?*

*R: That I don’t know because some of fever patients are not going to health centre. If there is VMW in village, they will come to volunteer. They don’t like going to health centre because they don’t want to wait. Here in village is quick for them if he has malaria after test, we provide treatment. It is much easier than going to health centre, at health centre they have schedule…. it takes a long [time], and no schedule here in the village [so, its] easier!*

*SSI-3*

While VMWs progressively developed the knowledge and skills necessary to smoothly use the biosensor machine over weeks and months, most of the VMWs reiterated the need to provide more training on the machine, its use, and interpretation of results. There were progressive improvements in skills and capacity to use the biosensor, which were corroborated by the observation-based questionnaire collected by the training supervisors.

**Fig. 6:**
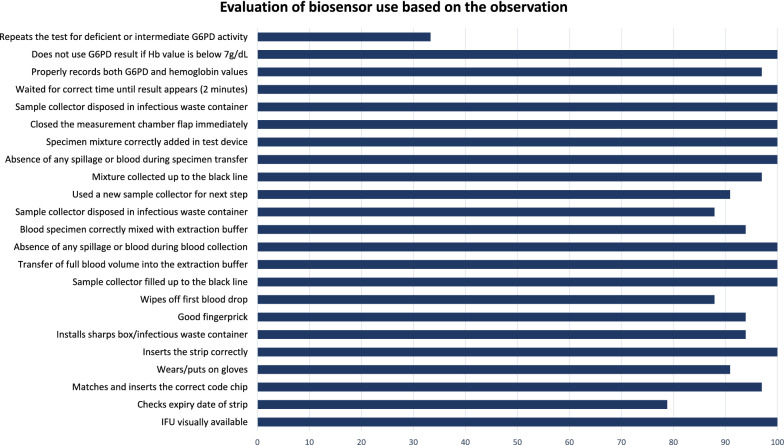
Evaluation of biosensor use by health workers based on the observation by trainer at M8

During the observation, 26 out of 33 (79%) participants checked the expiry date of the test device. VMWs were asked to record their reading of G6PD activity levels exactly how they were doing it during their routine use of the biosensor. Unless there were obvious errors (e.g. display of error codes such as E-2 or n-A, or complete blackouts), VMWs did not repeat tests in case of intermediate (2–6 U/g Hb) or deficient G6PD (< 2.0 U/g Hb) readings as they were instructed to record the first readings. Only 11/33; 33% of the participants repeated the test for deficient and intermediate G6PD activity, mostly for their own practice. All other items checked as part of the performance assessment were completed satisfactorily (Fig. 6).

Some VMWs shared their specific confusion for example having to use two pipettes (Ezi-tubes). When prompted, VMWs spontaneously attributed such procedural failures to their own forgetfulness and thus stressed the need for more (repeated) trainings.

### Suggested improvements

Apart from appreciating regular training and supervision, there were practical challenges in using the biosensor which were mostly related to the necessary supplies (e.g., buffer, chip codes and Ezi-tubes), the machine, and their usage (Table 3 and Fig. 5). VMWs expressed concerns around the size of the Ezi-tubes, reasons for using two, and the need to be precise in using them. Any deviation from these steps were likely to result in errors. For instance, retention of bubbles in the blood sample and use of insufficient or excess buffer solution produced erroneous readings. The sample processing steps also required promptness. For instance, leaving the ‘measurement chamber flap’ open longer than recommended (it should be closed immediately) could lead to an error.

**Table 3:** Concerns and recommendations around the use of biosensor machine

Items	Problems	Data
Pipette	Black line in pipette is hard to appreciate. Especially older VMWs had problem appreciating the black line Pipettes are too small. When the pipette takes bubble, it can affect the result Need to use two pipettes	*R: Using two pipettes is difficult…..and take other new one for sample and put into machine I feel difficult and my hands were shaking because that hole is small, I have to be very careful and very concentrate to the hole, that is the difficulty. If possible, we use one pipette, it can be confusing when it looks same as the previous. SSI-15* *I: If there is bubble inside and you still put into machine, for example. Do you think you get a good result or not?* *R: No… teacher, if the amount [is] low or over, it will be [show an] error. SSI-14*
Sample Processing	The sample processing steps require a prompt completion, letting the measurement chamber flap open for little longer, machine will display an error	*R: Which aspects do you think it is easy to use for the biosensor? I: In total, its easy …..when we take the sample…[but]…ooh not easy sometime if sample taking is more or over, and the easy aspect is battery.….if the sample is enough the result [will] appear, and if the cap [remains] open for longer time it [result] will not appear, It is an error. And sometimes it [reading] came with difference that’s what I did not want. It’s error. I: yes. SSI-3*
Test device	The hole within the test device is too small and sometimes mixture of buffer and blood were not able to be placed in it	*I: What about one hole inside when you put the sample whether it gets to the side of that hole?* *R: There was a little, but I did not clean because I am scared of [getting] it broken* *I: Do you think it is too small?* *R: It is too small, sometime the sample is poured out to the side of the hole* *I: That’s why it showed the different result due to the sample which was not enough. SSI-10*
Chip code	Insertion and removal of chip code is difficult. Removing chip code was difficult, some used knives to remove it and were scared that it could break	*I: Ohm….I heard someone told me about its difficulty, oh… yes at XXX village…VMW, she tried to remove it with the end of the sharp knife but it did not come out, so she decided to keep it in there. SSI-15* *I: Given you have used in the field setting, would you like to share with us, how the challenges you have identified can be avoided for future use?* *R: It is hard to remove the chip code out after each test, I think it would be better [to leave it], I left it inside, no need to take out and batteries as well. SSI-10*
Buffer tube	VMWs did not feel comfortable with buffer tube because there is nothing to support it, it can fall down any time by wind, or air from fan. It should have something to support; support for buffer tube so that it would be easy while processing	*R: I don’t like it that it was made without stand to support, each time my children helped by standing nearby to hold it for me when I processed, because I was worried about [it] falling down. SSI-20* *R: It should be harder than this [kit box], and the one we are using today it is easy to tear because we cannot finish tests in a month. I like buffer but I don’t like that it doesn’t have the support, for me I asked my husband to drill a small piece of wood with a hole to stand the buffer tube when I process the G6PD for patients. SSI-21* *R: No matter with me, but if it can be made any plastic with hole to support the buffer avoiding somehow falling down while we process the sample, that would be great. I made myself [one] from wood with drilling a hole to support the buffer. SSI-13*
Device package box	The device package box is made from paper and thus can absorb water and is vulnerable to easy damage	*I: What do you think about packing of the box, and test?* *R: I also want the box to be updated to harder or [made] from plastic, mine already broken because I travelled to forest, if I am not going to forest no matter with it. SSI-30* *I: What about the box of test?* *R: It is a kind of paper easy to get damaged, if it can be the plastic, it would be great so that it won’t absorb water,… Sometimes when I am visiting patient’s house and rain falls it will get wet, who knows? SSI-15*
Battery	There is no notification about battery and VMWs could not identify the status of the battery. Electricity-chargeable battery would have been great	*I: What do you not like about the biosensor?* *R: There was nothing that I did not like it, it does not have any weak points, and its easy to use, only the battery that we cannot know the [status of] power remaining or not. SSI-32*

Pipetting the buffer mixed blood sample into the test device was found to be difficult because of the small size of the sample receptor hole (sample well) in the test device. Some of the VMWs, particularly older ones found it difficult, perhaps related to poor eyesight, to place the specimen within the confines of the sample well. There were instances where samples could not adequately be placed, leading to erroneous readings, including mostly blank screens and error codes (n-A or E-2).

VMWs also shared their experience related to the parts of the machine that indirectly had an impact on sample processing. For instance, inserting the chip code is an essential step in the process, but its insertion and removal was found to be difficult. The buffer tube’s shape, particularly the lack of a wide base for the buffer tube, which would allow it to stand on its own, was a concern in field settings without lab benches. Many VMWs complained that the buffer tube not being able to remain upright led to the buffer spilling out. To address this problem, some VMWs built makeshift stands out of wood to hold the buffer tube.

At present, the device package box is made out of paper. The paper boxes contain all the essential supplies required for the tests. VMWs shared their experience of using these ‘paper made kit boxes’ in the field setting which were destroyed by water (rain), sweat, and repeated handling. VMWs often used plastic bags to protect the kits from rain and recommended plastic boxes for the future. There were also concerns around the devices’ batteries such as lack of notification of battery status (akin to the battery percentage displayed in the phone-screens), or VMWs were not able to appreciate the low battery alert. VMWs recommended a mechanism for battery status notification and suggested replacing the current batteries with electricity-chargeable batteries.

### Recommendations for implementation of G6PD testing and radical cure

VMWs appreciated the geographical barriers that prevent malaria patients from seeking care at health centres, particularly for those villages which are far away from the health centres. VMWs strongly recommended the community management of vivax malaria through deployment of the biosensor and provision of radical cure in the village instead of patients having to travel to the health centre (also echoed during post-study engagement with the participants). Often these patients fail to travel to health centre and seek care from community-based VMWs.

*I: What would you suggest policymakers/CNM about its use? Do you think this device can be added into daily routine of VMWs?*

*R: I would suggest that the machine should be provided to VMWs in village [which would be] better for all VMWs because some patients are from remote area, and they don’t have gasoline to drive to health centre. If we can use the radical cure treatment in village [that would be] is the best, it is near for patients, … I think if we have machine, medicine and training they can treat in village.*

*SSI-19*

VMWs also stressed the need for repeated training and supervision when they are devolved with the responsibilities for community-based management of vivax malaria. All VMWs felt that new community health workers could not use the biosensor without training.

A few VMWs were explicit in recommending the malaria control programme to allow VMWs to use primaquine for radical cure. The most frequent reasons for such recommendations were again embedded in barriers related to the accessibility of health centres. In the absence of an adequate treatment for vivax malaria in the community, they must refer patients to health centres, which requires overcoming several barriers and was not popular among community members.

*I: Let them [patient] drive motor bike first to health centre, for example if patient is living xxxx or yyyy villages [the furthest villages]*

*I: That’s our challenge here in villages, and I want to suggest organizations or national level [CNM] to help us to have this machine in every villages and have primaquine [single dose PQ with AMQ] such as before but in more doses…….now at the national level [they] changed this system and do not allow VMWs to have primaquine,….only AMQ for first dose, there is no primaquine, the patients have to go to health centre, so it is not easy for the far people [far from health centre].*

*SSI-6*

## Discussion

In the context of rapidly declining falciparum malaria and a relative increase in vivax malaria, VMWs serve as the structural units of community malaria (health) services. To achieve malaria elimination by 2025 [2], it may be important to train and enable community-based health workers to manage vivax malaria appropriately at the first point of contact with the health care system.

### Integrating the biosensors in VMWs’ routine work

Although VMWs have been involved in diagnosis of vivax malaria since 2021, much of their work is limited to diagnosing vivax malaria and then referring patients to health centres for G6PD measurement and radical cure therapy. At the community, their responsibility is to monitor adherence to radical cure regimen and clinical supervision. This development has shrunk the roles and responsibilities of the VMWs, their usefulness for the community and consequently their standing in the community [34]. Such a reduction in responsibilities neither seem to serve community members well nor VMWs, and not even health centres who have to manage an increased workload. The spread of responsibilities can result in poor performance by each of these stakeholders ultimately affecting patients who are reluctant to travel to health centres. VMWs’ enthusiasm to include biosensor into their routine work was motivated by their desire to re-establish their roles and usefulness in the community in line with a previous study in Cambodia [25]. There were both tangible and intangible achievements attached to opportunities to use biosensors by the VMWs. For instance, being able to test G6PD in the community and potentially provide radical cure treatment in future were seen as an opportunity to re-define and rescue their declining roles [35]. Embedded within such roles were also opportunities and privileges to build good relationship, trust, and gain respect from community members. VMWs’ request to integrate the biosensor into their routine work was also motivated by their knowledge of the shortcomings of current referral system in which attrition of patients remains high [34].

This study offered VMWs to use the biosensor at the community followed by training and continuous support. A previous qualitative study from Bangladesh echoed the critical role of training and supervision before being able to integrate the biosensors in end-users’ current roles and responsibilities [23]. A multi-country study that assessed the usability of biosensors based on the questionnaire used in this study (Additional file 1) also highlighted the usefulness of such an assessment in informing the areas that need further training and supervision. For instance, few participants found categorizing G6PD readings (such as 4.5 and 9.2) to be difficult. Specifically recalling the intermediate range of G6PD for participants (without a visible reference) seem to have affected their interpretations. Consistent with the previous studies [22, 23], this study provides further evidence that adding the biosensor into VMWs’ routine armamentarium requires regular training, supervision, and support.

### Suggested improvements

The majority of recommendations made by VMWs emerged from their day-to-day interaction with the biosensor and have relevance to improving the currently available biosensors. The recommendations for improvements emerged from operating the biosensors for more than a year in the rural communities [22, 23]. Issues such as replacing the paper box with a plastic box, designing a buffer holder, smoothening the chip code insertion, improving battery notifications (not just when it is low) and replacing standard batteries with electricity-chargeable batteries (Table 3) bear high relevance to all the stakeholders. These findings thus are practicable, translatable, and may critically guide improvements to redesign the next generation biosensors.

### Implications for implementation of radical cure regimen

WHO recommends four ‘As’, availability, accessibility, appropriateness and affordability for a medical product/device to be optimal [36]. Some of the components of four ‘As’ are explored in this study, for example, appropriateness of the biosensor use by VMWs in the community [37]. The relevance of appropriateness is even more important for point of care (POC) medical devices, particularly to assess their functional abilities under field conditions that can emerge from device break down, poor design, lack of maintenance, quality control and end-users’ skills. Up to three-quarters of medical devices do not function in novel settings and remain unused [36, 37].

In this study, the majority of VMWs recommended the deployment of biosensors in the community in contrast to current availability of the biosensor machine at the health centre only. Their motivations for such recommendations were underpinned by their progressive development of skills and capacity to use biosensor and the reluctance of patients to travel to health centres for G6PD testing and radical cure treatment.

Community management of vivax malaria is a promising strategy as Cambodia embarks on the ambitious goal to eliminate malaria by 2025. With malaria receding to communities which are located at the forest fringe and remote areas, far away from the health centres, community members are unlikely to visit health centre for the radical cure treatment, they may rather seek symptomatic treatment with VMWs or locally available alternative health services [38–44]. Reluctance to travel to health centre also can lead to poor access (and adherence) to radical cure and persistence of a vivax malaria reservoir. The roll out of the radical cure may be particularly urgent in forest and forest fringe areas in response to malaria among forest goers in Cambodia and is in line with WHO’s focus on community focused interventions for the ‘last mile’ in malaria elimination [45].

It is critical to ensure that community members receive radical cure at the point of first contact, rather than obliging patients with limited resources to travel extra miles to reach health centres.

### Strengths and limitations

The study was well-resourced in terms of logistics supplies, staff for training, supervision (monthly calibration, and control testing of the biosensor), and evaluation which may be different when rolling out biosensor deployment at programmatic level. Multiple methods were utilized to assess how VMWs were integrating biosensor in their daily routine, and this allowed triangulation of findings necessary for robust conclusions. Although VMWs used biosensors among an adequate number of vivax (n = 63) and febrile patients (n = 1077) for the practice, their accuracy of usage, and the reliability of readings are beyond the scope of the current study. An ongoing sub-study of RORC will compare the readings between VMWs and lab technicians to a reference method.

Providing adequate tests to VMWs was part of the training in this study, but with the current decline in vivax malaria incidence, VMWs may struggle to maintain their skills necessary for its use. Patients’ perceptions and experiences related to the use of the biosensor was only reported indirectly through VMWs. Future studies could also explore patients’ perspectives around biosensors. VMWs were also convened to provide feedback to the current findings as a part of post-study engagement. During these two meetings, VMWs confirmed that traveling to health centres monthly for meetings, training, calibration and control test for the biosensor was not a burden. In addition, VMWs expressed their altruistic motivations to serve in the community when asked about compensation in potential future programmatic deployment.

Quantitative assessments of VMWs’ skill sets are limited by the relatively small numbers of participants (n = 33) recruited in this study, which poses limitations in conducting a robust statistical analysis and drawing conclusions. The study took place in villages in Pursat province and may be generalizable to similar settings within Cambodia. However, the epidemiology and management of vivax malaria vary between countries, and even within Southeast Asia, and therefore some caution is needed in generalizing the findings of this study to community health workers outside of Cambodia. Qualitative and quantitative assessments, including observations, occurred at various timepoints which offered us some details of the operational development over time and thus complementing to each of these methods. For instance, presenting only a post-training questionnaire assessment as an evaluation of how VMWs perform with the biosensor would have offered a narrower picture compared to current study which provides a broader and more realistic evaluation of their skills and the feasibility of deploying biosensors in the future.

## Conclusion

VMWs were provided training and supervision (on a monthly and on an ad hoc basis) and were able to use the biosensors appropriately as demonstrated by progressive development in their skills and enthusiasm for using the tests. They showed a high level of motivation to take on additional responsibilities necessary for the community management of vivax malaria. Apart from the functional contribution of biosensors in potentially allowing VMWs to offer radical cure, critical for the elimination of malaria in Cambodia by 2025, such additional skills and integration of biosensor into their routine work were seen as opportunities to redefine their roles and usefulness in the community. With the multitude of barriers to attend health centres it is critical to build the capacities of village-based community health workers to diagnose malaria, assess G6PD status reliably, and provide radical cure under the supervision of health centres.

## Supplementary Information

**Additional file 1: **Post-training questionnaire.

**Additional file 2: **Observation based quantitative questionnaire at the community setting.

**Additional file 3: **Semi-Structured Interviews (SSIs) Guide.

## Data Availability

The data is available upon request to the Mahidol Oxford Tropical Medicine Research Unit Data Access Committee (http://www.tropmedres.ac/data-sharing) complying with the data access policy (http://www.tropmedres.ac/_asset/file/data-sharing-policy-v1-0.pdf).

## Abbreviations

COVID-19: Corona virus infectious diseases-2019
CNM: National center for parasitology, entomology and malaria control
G6PD: Glucose 6 phosphate dehydrogenase
G6PDd: Glucose 6 phosphate dehydrogenase deficiency
MMWs: Mobile malaria workers
POC: Point of care
RDT: Rapid diagnostic test
RBCs: Red blood cells
RORC: Roll out radical cure
SSIs: Semi-structured interviews
USFDA: United states food and drug administration
VMWs: Village malaria workers
WHO: World Health Organization
