# Supplementary material for: Glucose 6 Phosphate Dehydrogenase (G6PD) quantitation using biosensors at the point of first contact: a mixed method study in Cambodia
Source: Malar J. 2022 Oct 4;21:282. doi: 10.1186/s12936-022-04300-9 (PMC9531219; doi:10.1186/s12936-022-04300-9)
Supplement: Supplementary file 1 — Additional file 1: Post-training questionnaire. [file 12936_2022_4300_MOESM1_ESM.pdf]

APPENDIX A: BIOSENSOR ASSESSMENT (Developed by PATH for use in user proficiency assessments for STANDARD G6PD studies, 2019)

Label comprehension and results interpretation questionnaire

This questionnaire is intended to assess the user's ability to understand the product label and instructions for use and interpret the results. For the results interpretation component of the assessment, the trainer should use local ranges for normal, intermediate, and deficient or the ranges in the product instructions for use.

UNIQUE ID:

**Circle the correct answer for multiple choice questions.**		
**N**	**Questions**	**Options**
1)	The STANDARD G6PD test can be used to identify people who have G6PD deficiency	1. Yes<br>2. No
2)	What does the STANDARD G6PD test measure?	1. Presence of antibodies *(antibodies are our fighter cells against malaria)*<br>2. Presence of *malaria parasite*<br>3. Enzyme reaction *(A substance required for the body to function)*<br>4. None of the above
3)	What is the operating temperature range for test operation?	1. 2°–30°C<br>2. 15°–40°C<br>3. Must be performed in an air-conditioned lab<br>4. 0°–40°C
4)	The SD Biosensor STANDARD G6PD test can be used with which types of samples?	1. Capillary blood *(blood from finger prick)*<br>2. Venous blood *(blood drawn using a syringe)*<br>3. Plasma *(clear part of blood without red cells)*<br>4. Capillary and venous blood<br>5. All the above
5)	How do you use the code chip?	1. The number of the chip and the number printed on the test strip pouch must match.<br>2. The code chip is not really necessary.<br>3. The same code chip can be used all the time.<br>4. You need to change the code chip every time you run a new test.

6)	When do you insert the test strip into the analyzer?	1. Add the sample to the test strip first, and then insert it into the analyzer.<br>2. The test strip remains inserted in the analyzer between samples.<br>3. After the code chip is entered into the analyzer and before the sample is collected and applied.<br>4. It doesn't matter when the test strip is inserted into the analyzer.
7)	How do you mix the blood sample and the buffer?	1. Add the blood sample to the buffer and mix with the EziTube by pressing and releasing the tube 10 times.<br>2. Add the blood sample to the buffer and shake 10 times.<br>3. Put the blood sample in another container and pour in the buffer.<br>4. The blood sample and the buffer should not be mixed.
8)	After mixing the buffer and the sample, how much of the mixed specimen should be added to the test strip?	1. All of the mix should be added to the test strip.<br>2. It doesn't matter how much, as long as some is placed on the strip and the test will run.<br>3. Use a micropipette to put 20 microliters on the test strip.<br>4. Use 10 microliters. The black line on the EziTube is the right amount.
9)	After mixing the sample and buffer, how long should you wait before applying the mixture to the test strip?	1. Apply after 2 minutes.<br>2. Apply after 5 minutes.<br>3. Apply immediately.<br>4. It can be applied at any time.
10)	How many Ezi Tubes do you need to run one sample?	1. 0<br>2. 1<br>3. 2<br>4. 3
11)	Can you reuse the test strip?	1. Yes<br>2. No
12)	How can you avoid any injury caused by this test?	1. Use gloves<br>2. Do not ingest<br>3. Discard the used test devices according to the local guidelines<br>4. All of the above
13)	Which G6PD result are you most likely to see if your patient has very low G6PD activity and is G6PD deficient?	1. 13.6 U/ gHb<br>2. 1.2 U/ gHb.<br>3. Error message.<br>4. 9.7 U/ gHb

Please read the following test results, record the results, and identify how you would interpret the result.

<table>
<tr><th>Number</th><th>Result</th><th>Record</th><th>Interpretation—select one
1. Normal
2. Intermediate
3. Deficient
4. Test did not work (error or NA)</th></tr>
<tr><td>14</td><td>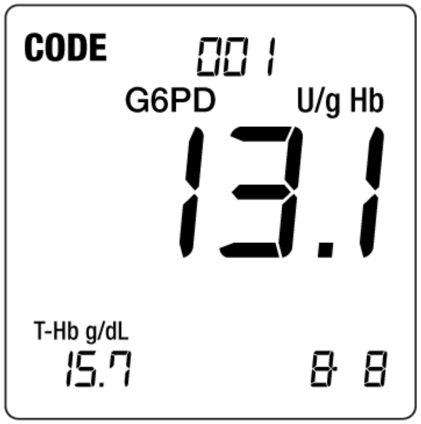
</td><td>G6PD U/g Hb ___________

Hb g/dL ___________</td><td></td></tr>
<tr><td>15</td><td>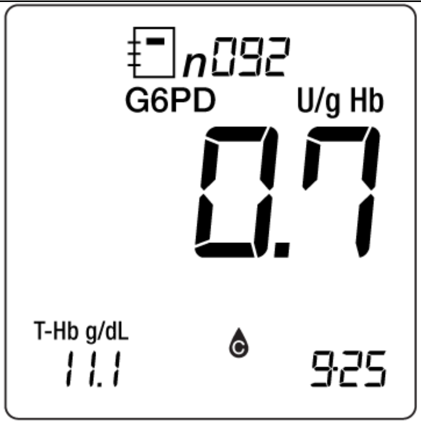
</td><td>G6PD U/g Hb ___________

Hb g/dL ___________</td><td></td></tr>
</table>

16	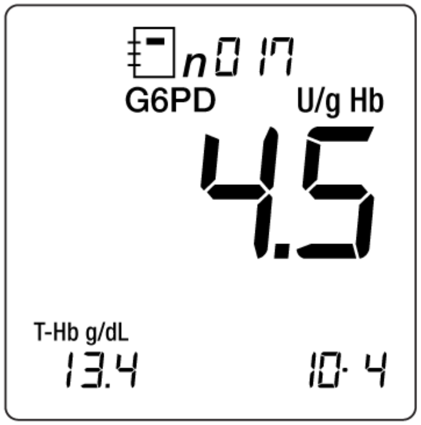	G6PD U/g Hb ___________<br><br>Hb g/dL ___________	
17	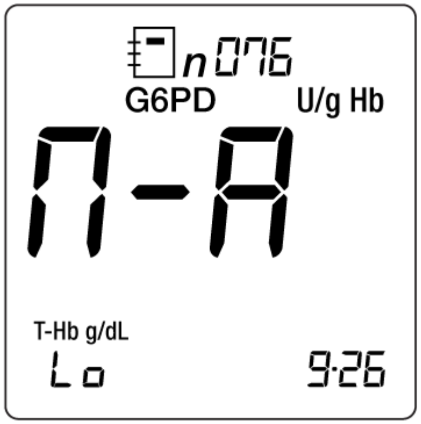	G6PD U/g Hb ___________<br><br>Hb g/dL ___________	
18	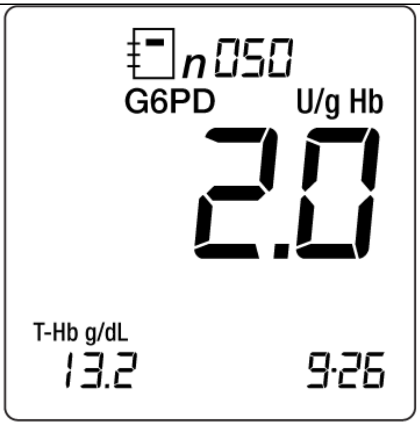	G6PD U/g Hb ___________<br><br>Hb g/dL ___________	

19	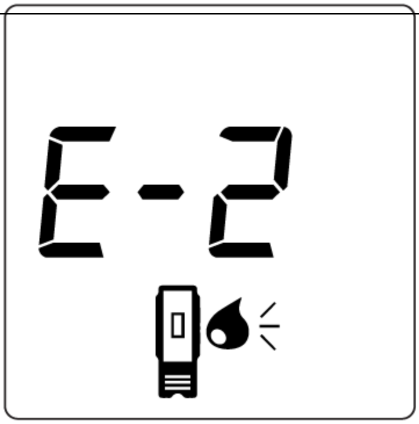	G6PD U/g Hb ___________<br><br>Hb g/dL ___________	
20	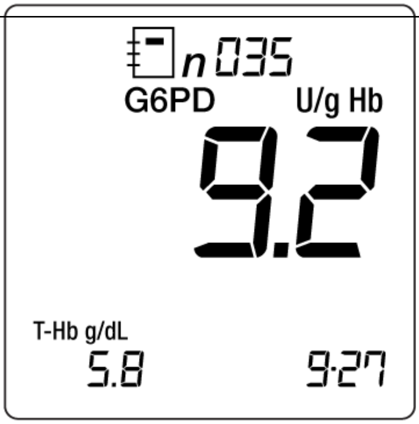	G6PD U/g Hb ___________<br><br>Hb g/dL ___________	
