# Supplementary material for: Glucose 6 Phosphate Dehydrogenase (G6PD) quantitation using biosensors at the point of first contact: a mixed method study in Cambodia
Source: Malar J. 2022 Oct 4;21:282. doi: 10.1186/s12936-022-04300-9 (PMC9531219; doi:10.1186/s12936-022-04300-9)
Supplement: Supplementary file 2 — Additional file 2: Observation based quantitative questionnaire at the community setting. [file 12936_2022_4300_MOESM2_ESM.pdf]

APPENDIX B: Ease of use and acceptability of the STANDARD™ G6PD analyser in POC settings by FIND. Proficiency assessment of Health Workers V1.0, 2nd October 2019

1.	Health worker ID:	____________________
2.	The assessment was carried out by:	__________________________
3.	Role:	_____________________________
4.	Telephone:	Trainer's telephone number______________
5.	E-mail:	Trainer's email_________________________
6.	Date (dd/mm/yy):	______________________________
7.	Name of Health Facility:	_______________________
8.	Village:	____________

Brief Assessment Instructions:

- Instruct the Health Worker (HW) to do the test on voluntary members of the study team (assessment conducted immediately after the training).
- Before starting the assessment, read through the questionnaire again to be familiar with all questions and have in mind which key steps will have to be observed.
- While the HW conducts the test, observe carefully each of the key steps, and quickly answer the relevant questions in the questionnaire. Provide comments wherever needed or useful, e.g. describe any difficulties encountered by the HW, or note any comments he/she might make while conducting the test.
- At the completion of the assessment, re-read all your answers to check the form is complete, and clarify any comments as required.

Abbreviations:

G6PD	Glucose-6-phosphate dehydrogenase
Hb	Hemoglobin
HW	Health Worker
IFU	Instructions for use
ID	Identification number

Information about the HW: Ask the HW the following questions			
**S.N.**	**Questions**	**Answers**	**Comment(s)**
9.	Age	________Years	
10.	Sex	1. Male 2. Female	
11.	Main professional activity<br>(can check more than one box):	1. Laboratory testing<br>2. Blood collection<br>3. Medical consultation<br>4. Laboratory management<br>5. Other (specify in comments section)	
12.	Education/diploma:	1. Lab technician<br>2. Nurse<br>3. Lab scientist<br>4. Medical Doctor<br>5. Other (specify in comments section)	
13.	Professional experience	1. More than 10 years<br>2. 5-10 years<br>3. 1-5 2years<br>4. Less than 1 year	
14.	Prior participation in research studies?	1. Yes, more than 2 studies<br>2. Yes, but only 1-2 studies<br>3. None<br>4. Other (specify in comments)	
15.	**If yes to the above**, what was your role in this research study/ies<br>(can check more than one box)?	1. Patient screening/enrolment<br>2. Sample collection (blood etc.)<br>3. Patient testing<br>4. Patient consultation/treatment<br>5. Laboratory analyses<br>6. Assistance role only (no direct involvement in patients or samples management)<br>7. Other (specify in comments)	
16.	Prior experience with the STANDARD™ G6PD test:	1. Yes, more than 1 year<br>2. Yes, but less than 1 year<br>3. None<br>4. Other (specify in comments section)	
17.	Prior experience with similar tests	1) Yes, more than 1 year	

	(e.g. Hemocue, Biosensors,..):<br>*Please specify which in the comments*	2) Yes, but less than 1 year<br>3) None<br>4) Other (specify in comments section)	
18.	Additional comments:		
	**Assessment questions:**		
	**Q1: Set-up for testing**	**Answers**	**Comment(s)**
19.	Has the IFU visually available:	1. Yes<br>2. No	
20.	Checks expiry date of strip:	1. Yes<br>2. No	
21.	Inserts the correct code chip (matching code on test pouch):	1. Yes<br>2. No	
22.	Wears / puts on gloves:	1. Yes<br>2. No	
23.	Inserts the strip correctly (correct orientation, fully inserted):	1. Yes<br>2. No	
24.	Installs sharps box / infectious waste container:	1. Yes<br>2. No	
25.	Additional comments:		
	**Q2**: Blood collection and G6PD testing:	**Answers**	**Comment(s)**
26.	Good fingerprick<br>(firm stab, large blood drop):	1. Yes<br>2. No	
27.	Wipes off first blood drop:	1. Yes<br>2. No	
28.	Adequate blood volume picked (sample collector filled up to the black line):	1. Yes<br>2. No	
29.	Transfer of full blood volume into the extraction buffer:	1. Yes<br>2. No	
30.	Absence of any spillage or contact of blood with health worker's gloves, skin, clothing or any other surface during the blood transfer:	1. Yes<br>2. No	
31.	In case of spillage, correctly cleans and disinfects the contaminated area / changes gloves or clothing:	1. Yes<br>2. No<br>3. NA	
32.	Blood specimen correctly mixed with extraction buffer (press-release 8-10 times):	1. Yes<br>2. No	
33.	Sample collector disposed of in infectious waste container or sharps box:	1. Yes<br>2. No	
34.	Used a new sample collector for next step (no re-use of previous sample collector):	1. Yes<br>2. No	

35.	Adequate volume of mixture collected with new sample collector (filled up to the black line):	1. Yes<br>2. No	
36.	Absence of any spillage or contact of blood with health worker's gloves, skin, clothing or any other surface during the specimen transfer:	1. Yes<br>2. No	
37.	Specimen mixture correctly deposited in specimen application well<br>(entire volume on the filter pad):	1. Yes<br>2. No	
38.	Closed the measurement chamber flap immediately:	1. Yes<br>2. No	
39.	Sample collector disposed of in infectious waste container or sharps box:	1. Yes<br>2. No	
40.	Waited for correct time until result appears (2 minutes):	1. Yes<br>2. No	
41.	*If using G6PD controls:*<br>Properly reconstitutes the controls and prepares them for analysis:	1. Yes<br>2. No<br>3. NA	
42.	*If using G6PD controls:*<br>Successfully performs the assay with the controls and obtains 'in range' values:	1. Yes<br>2. No<br>3. NA	
43.	*If using G6PD controls:*<br>Demonstrates reproducibility by obtaining similar values upon repeating the assay:	1. Yes<br>2. No<br>3. NA	
44.	Additional comments:		
	**Q3: Test result reading & interpretation**	**Answers**	**Comment(s)**
45.	Properly records both G6PD and hemoglobin values:	1. Yes<br>2. No	
46.	Does not use G6PD result if Hb value is below 7g/dL (note NA in comments section if Hb value is above 7g/dL)	1. Yes<br>2. No	
47.	Repeats the test if result indicates deficient or intermediate G6PD activity:	1. Yes<br>2. No	
48.	Additional comments:		
