# Supplementary material for: Glucose 6 Phosphate Dehydrogenase (G6PD) quantitation using biosensors at the point of first contact: a mixed method study in Cambodia
Source: Malar J. 2022 Oct 4;21:282. doi: 10.1186/s12936-022-04300-9 (PMC9531219; doi:10.1186/s12936-022-04300-9)
Supplement: Supplementary file 3 — Additional file 3: Semi-Structured Interviews (SSIs) Guide. [file 12936_2022_4300_MOESM3_ESM.pdf]

APPENDIX C: Semi-structured interviews with VMWs on the practicalities of biosensors

Study title: Quantitative G6PD diagnostics followed by treatment decision for vivax malaria: how do village malaria workers perform?

Semi-structured interviews

(These are only guides to prompt interviewer and interviewee to adhere to the topic. Please feel free to add the themes and concerns as you see fit)

AUDIO FILE NAME:

Introduction (read out):

I am …………………… from Mahidol-Oxford Tropical Medicine Research Unit (MORU) based in ………., Cambodia. I am a researcher and I am approaching you to discuss on practicalities of using biosensor in your routine work. As you know diagnosing G6PD enzyme deficiency is essential to inform the decision for radical treatment of vivax malaria, I would like to explore your experience with biosensor in your routine work. I am interested to know the ease of using the biosensor, interpretation of the results and informing treatment for vivax malaria. Your experience and perspectives will guide us and inform the future decision on feasibility and practicalities of deploying biosensors at the field level in Cambodia and outside. There are no direct benefits attached to your participation in this interview. I will respect your time and if you feel uncomfortable or distressed due to questions asked or the time consumed, you can inform me at any time and I will immediately stop the interview. Also, you can drop out of the interview at any time and you do not have to give me reasons for it. If you consent to participate, I would like to inform you that this interview will be anonymized, the information you will provide will be confidential. Please feel free to ask questions related to this study at any time before making a decision. If you agree, I would like to audio-record this interview for transcription and analysis.

SECTION-I: General Information

**Socio-demographic characteristics of respondents**	1. Age		
	2. Gender	Male ☐ Female ☐ Other ☐	
	3. Workplace	Health centre ☐	Village ☐ Name______________
	4. Qualification		
	5. Biosensor machine ID		

SECTION-II: Biosensor and its use

**Themes**	**Guide**
**Characteristics of VMW's work without biosensor**	• How are you doing with your job?<br>• How many fever patients do you see in a month?<br>• How many cases of vivax malaria do you see in a month?<br>• How do you normally treat vivax malaria patients?<br>• Do you provide radical cure treatment? (Regimen for radical cure)<br>• Do you use G6PD test before providing radical cure treatment?

<table>
<tr><td></td><td>What do you use for G6PD test? How do you interpret?</td></tr>
<tr><td>Addition of biosensor into VMWs' work</td><td>How are you doing with the biosensor?
How long have you been using the biosensor?
Do you feel that you have received adequate training on how to use the biosensor? (prompt: what aspects of training was useful? What more would you like to be trained in future?)
Can you talk about how are you finding this in your everyday work? (prompts: ease of use, technical difficulties, probabilities, strips, battery, safety, water resistance, rainy seasons, time taken for test…..)
Do you use it among all vivax malaria patients?
Have patients commented on the device? (prompt: questions on why do you use it? Time consumed, extra blood test, unsure of its purpose, ……)
Overall, do you find biosensors (well) integrated in your work? (have you accepted them or are they still burden in your work?) Why?</td></tr>
<tr><td>Product improvement</td><td>What do you like about the biosensor?
What do you not like about the biosensor?
What do you think should be improved with the current biosensor?
Use following prompts:
With the machine
With the procedures
With the way you receive buffer, strips, and machine
i.e. Sufficient pipettes
What do you think about the packaging?
Of a new box of strips
Packaging of pipettes
Sealing of buffer</td></tr>
<tr><td>Use of biosensor in future</td><td>Do you think biosensor can be used by any other VMW <u>with or without</u> a one day standardized hands-on training?
Given you have used in the field setting, would you like to share with us, how the challenges you have identified can be avoided for future use?</td></tr>
</table>

	• What would you suggest other (untrained) VMWs on its use, ease, practicalities?<br>• What would you suggest policymakers/CNM about its use? Do you think this device can be added into daily routine of VMWs? (or do they suggest it to be only used at the health centre/or by lab personnel?)
**Free opinion**	Would you like to share with us any free opinion that I may have missed discussing with you?
The End	
